# Association between work-related health problems and job insecurity in permanent and temporary employees

**DOI:** 10.1186/2052-4374-25-15

**Published:** 2013-09-11

**Authors:** Won-Wook Lee, Jae-Bum Park, Kyoung-Bok Min, Kyung-Jong Lee, Min-Su Kim

**Affiliations:** 1 Department of Occupational and Environmental Medicine, Ajou University Hospital, Suwon, South Korea; 2 Department of Occupational and Environmental Medicine, Ajou University School of Medicine, Suwon, South Korea

**Keywords:** Job insecurity, Work-related health problems, Permanent workers, Temporary workers, Korean working conditions survey

## Abstract

**Objectives:**

This research was conducted with an aim of determining the correlation between job insecurity and an employee’s work-related health problems among permanent and temporary workers.

**Methods:**

Using the data from the First Korean Working Conditions Survey conducted in 2006, a total of 7,071 workers, excluding employers and the self-employed, were analyzed. Work-related health problems were categorized as backache, headache, abdominal pain, muscular pain, stress, fatigue, insomnia, anxiety or depression. Each problem was then analyzed for its relationship to job insecurity through logistic regression analysis.

**Results:**

Among the 7,071 workers, 5,294 (74.9%) were permanent workers and 1,777 (25.1%) were temporary workers. For the permanent workers, presence of high or moderate job insecurity appeared more closely linked to backache, headache, abdominal pain, muscular pain, stress, fatigue, insomnia, anxiety, and depression compared to absence of job insecurity. However, for the temporary workers, only depression appeared to be associated with the presence of high job insecurity.

**Conclusion:**

The study showed that the presence of job insecurity is correlated with work-related health problems. The deleterious effects of job insecurity appeared to be stronger in permanent than temporary workers. Additional research should investigate ways to effectively reduce job insecurity.

## Introduction

As a type of occupational stress, job insecurity has been defined in a number of ways, from concerns about maintaining a current job or work position, fear of total loss of any type of employment, to a degree of concern about the extent of job security [1-3]. All of these varying definitions are based upon the uncertain future and the individual experiences of workers [4]. Job insecurity, in effect, is a perception, and is different from the threat of losing one's job or the actual dismissal from the employment because, employees are apt to experience this with or without the true risk of such losses [3]. Even though it is generally known that temporary employees experience more job insecurity than do permanent ones, it is always possible for permanent employees, who are less likely to actually be dismissed from work, to perceive job insecurity as a threat throughout their employment just as temporary employees do, and because more employees are likely to experience such threat than actually experience dismissals, job insecurity has become an important occupational stress factor [4,5].

Within the most recent few decades, a pan-global economic crisis has caused many corporations and companies to carry out restructuring, mergers, etc., and this brought about massive layouts and expansion of temporary employment in the secondary labor market, which has exposed an increasing number of workers to the perception of job insecurity [6]. Hence, reports and studies generally identify such job insecurity as contributing negatively to the psychological and physical health and well-being of employees [1,3,7-12]. Up until now, studies in South Korea (hereafter, “Korea”), however, have treated job insecurity along the dame lines as actual job losses, threats of job dismissal, and/or temporary employment [13], and some have limited their subjects to a certain job group [10-12]. Furthermore, even though reports from other countries indicate different correlations between the health of the employees and the job insecurity according to different types of employment [14,15], research on how job insecurity impacts different types of workers has been sparse in Korea.

There are two hypotheses that have to do with the type of employment and job insecurity. The intensification hypothesis explains that the perception of job insecurity of temporary employees contributes to more health problems than permanent employees’ experience. On the other hand, the violation hypothesis explains that even though temporary employees experience more job losses and risks than permanent employees, permanent employees' perception of job insecurity causes more detrimental health issues [14,16]. In many studies outside of Korea, evidence has been gathered that supports the latter claim [15,17-19], but the results have only been found in some countries [14], and they were focused more on the psychological dimension [15]. Moreover, the research on both the physical and psychological health effects of job insecurity on workers did not show a significant association with job insecurity [20,21].

Our research, using the First Korean Working Conditions Survey (2006), aims to evaluate the hypothesis mentioned earlier, that job insecurity correlates to the health and well-being of employees, and goes on to determine the differences in work-related health and well-being according to the differing perceptions of job insecurity between two types of employees.

## Materials and methods

### Study population

Our research utilized the First Korean Working Conditions Survey, which was published in 2006. This survey was performed by the Occupational Safety and Health Research Institute (OSHRI), and was based on the Fourth European Working Conditions Survey. With an intent to set up all employees residing in South Korea as a cohort, the survey collected general information such as gender, age, lifestyle, and work environment factors such as occupation, area of specialty, type of employment, noxious exposure, and the shift system in order to acknowledge the extent of exposure of risk factors having to do with work environment and to provide appropriate information for policy. Paid employees from the age of 15 to 64 were included, and based on the 2005 Korea Population and Housing Census as a sampling frame, survey units were selected and trained surveyors visited each household to carry out the survey [22]. Out of a total number of 10,043 participants, with the exclusion of the self-employed, employers and those who did not complete with essential parts of the survey, 7,071 were selected as the study population. This study was reviewed by the institutional review board (IRB) of Ajou University Hospital.

### Study design

We have defined the group of participants with high job insecurity as those who have responded to the statement of the First Korean Working Conditions Survey (2006), “I will lose my job within the next 6 months” with “Agree” or “Very much agree”, those who reported themselves to be “Neutral” as a moderate job insecurity group, and those who answered “Disagree” and “Strongly disagree” as a low job insecurity group. Those who responded “yes “to the question “Have you ever had any hazardous accidents or health problems on account of your job?” and selected any one of backache, headache, abdominal pain, muscular pain (shoulder, neck, or upper or lower extremities), stress, fatigue, sleeping problems, anxiety, or depression as the health problem derived from the employment were defined as having work-related health problems. The participants who reported having one or more symptoms of backache, headache, abdominal pain, muscular pain, or fatigue were treated as a physical health problem group, and those with one or more of stress, sleeping problems, anxiety, or depression were considered to have psychological health problems. In addition, in order to determine the influence of hazardous exposure and work environment on the health of the employees, those who responded affirmatively to one or more of the queries on physical/chemical hazard exposure or ergonomic risk factor exposure for more than half of the working time were defined as an exposure group. The workers were also classified by contract type [22], into permanent and temporary employees. Permanent employees were defined as those who were employed full-time either indefinitely or with a limited contract period with a continuous working period of one year or more, and all the rest of the workers were classified as temporary employees.

### Statistical analysis

Our research was conducted, stratifying by employment type, to find the association between social demographic characteristics as well as work environment factors, on the one hand, and the level of job insecurity and work-related health problems, both physical and psychological, on the other. Then, adjusting for factors such as demographic characteristics and work environment, logistic regression was performed. Statistical analysis was done using Statistical Package for the Social Science (SPSS) for Windows version 19.0 (IBM Corp.: Armonk, NY, USA).

## Results

### General characteristics

The total number of employees included in the study was 7,071. Among them, 6,248 (88.4%) had low job insecurity, 446 (6.3%) moderate, and 377 (5.3%) high. Of the characteristics based on the different levels of job insecurity, the age of those with a high level of insecurity ranged from 25 to 54 years old, and those with low insecurity were mostly younger than 24 and over 55 years old. By gender, women had a higher sense of job insecurity than men. Those with lower job insecurity tended to have the group with a higher income, as well as a higher educational level. Regarding type of employment, the temporary employee group showed a higher level of job insecurity than the permanent ones did, with 11.6% and 11.1% reporting a moderate level and high level of job insecurity, respectively, while 4.5% and 3.4% of the permanent employees in the moderate and high level groups, respectively, reported job insecurity. The demographics also showed that physical laborers and those who were in the service industry had a higher incidence of moderate to high job insecurity than did those who were office clerks or professionals. Those who had moderate to high job insecurity were more likely to have more exposure to physical and/or chemical hazards or had higher ergonomic risk factors than not. Smoking, drinking, and the shift system, however, had no statistical significance among the different levels of job insecurity (Table 1).

**Table 1:** Characteristics of the participants

**Variables**		**All**	**Job insecurity N** ***(%)***			p-value*
			**Low**	**Moderate**	**High**	
Total		7,071	6,248 (88.4)	446 (6.3)	377 (5.3)	
Age group (years)						
	15-24	398	325 (81.7)	22 (5.5)	51 (12.8)	<0.001
	25-34	2,016	1,786 (88.6)	134 (6.6)	96 (4.8)	
	35-44	2,506	2,244 (89.5)	149 (5.9)	113 (4.5)	
	45-54	1,473	1,331 (90.4)	77 (5.2)	65 (4.4)	
	55-65	678	562 (82.9)	64 (9.4)	52 (7.7)	
Gender						
	Male	4,594	4,083 (88.9)	296 (6.4)	215 (4.7)	0.004
	Female	2,477	2,165 (87.4)	150 (6.1)	162 (6.5)	
Income (10,000 won/month)						
	<100	1,516	1,192 (78.6)	161 (10.6)	163 (10.8)	<0.001
	100-200	2,987	2,369 (88.3)	200 (6.7)	148 (5.0)	
	200-299	1,663	1,552 (93.3)	66 (4.0)	45 (2.7)	
	≥300	905	865 (95.6)	19 (2.1)	21 (2.3)	
Education completed						
	≤Middle school	1,024	846 (82.6)	100 (9.8)	78 (7.6)	<0.001
	High school	2,915	2,522 (86.5)	199 (6.8)	194 (6.7)	
	≥University	3,132	2,880 (92.0)	147 (4.7)	105 (3.4)	
Drinking (times/week)						
	No drinking	1,688	1,494 (88.5)	103 (6.1)	91 (5.4)	0.170
	≤1	3,084	2,722 (88.3)	182 (5.9)	180 (5.8)	
	≥2	2,299	2,032 (88.4)	161 (7.0)	106 (4.6)	
Smoking						
	Never	3,627	3,188 (87.9)	228 (6.3)	211 (5.8)	0.162
	Former	952	844 (88.7)	69 (7.2)	39 (4.1)	
	Current	2,492	2,216 (88.9)	149 (6.0)	127 (5.1)	
Contract type						
	Permanent	5,294	4,875 (92.1)	240 (4.5)	179 (3.4)	<0.001
	Temporary	1,777	1,373 (77.3)	206 (11.6)	198 (11.1)	
Occupation						
	Office clerk	1,448	1,324 (91.4)	66 (4.6)	58 (4.0)	<0.001
	Professional	1,486	1,377 (92.7)	60 (4.0)	49 (3.3)	
	Service industry	1,217	1,057 (86.9)	87 (7.1)	73 (6.0)	
	Physical labor	2,920	2,490 (85.3)	233 (8.0)	197 (6.7)	
Working hours per week						
	<35	571	442 (77.4)	71 (12.4)	58 (10.2)	<0.001
	35-44	2,649	2,413 (91.1)	124 (4.7)	112 (4.2)	
	≥45	3,851	3,393 (88.1)	251 (6.5)	207 (5.4)	
Work schedule						
	Non-shift	6,252	5,626 (88.4)	400 (6.4)	326 (5.2)	0.350
	Shift	819	722 (88.2)	46 (5.6)	51 (6.2)	
Physical & chemical hazards						
	Non-exposure	4,451	4,044 (90.9)	218 (4.9)	189 (4.2)	<0.001
	Exposure	2,620	2,204 (84.1)	228 (8.7)	188 (7.2)	
Ergonomic risk factors						
	Non-exposure	1,964	1,803 (91.8)	93 (4.7)	68 (3.5)	<0.001
	Exposure	5,107	4,445 (87.0)	353 (6.9)	309 (6.1)	

*Calculated by chi-squared test.

### Work-related health problems and the level of job insecurity by contract type

Work-related health was analyzed by stratifying participants into different contract types: temporary and permanent. The temporary employees reported more work-related health problems than did the permanent workers (data not shown). After the stratification, comparing work-related health problems at each level of job insecurity, it was found that permanent workers responded with more work-related health problems in higher level of job insecurity than those with low job insecurity. As for the temporary employees, even though there proved to be more complaints of work-related health problems compared to the permanent workers on the whole, unlike the permanent workers, the differences in work-related health problems by level of job insecurity were not statistically significant (Table 2).

**Table 2:** Results of work related health problems by contract type and level of job insecurity

**Variables**		**Permanent**			p-value*	**Temporary**			p-value*
		**Job insecurity**				**Job insecurity**			
		**Low**	**Moderate**	**High**		**Low**	**Moderate**	**High**	
All health problems	No	3,865 (79.3)	169 (70.4)	121 (67.6)	< 0.001	982 (71.5)	140 (68.0)	149 (75.3)	0.268
	Yes	1,010 (20.7)	71 (29.6)	58 (32.4)		391 (28.5)	66 (32.0)	49 (24.7)	
Physical health problems	No	3,925 (80.5)	170 (70.8)	124 (69.3)	< 0.001	994 (72.4)	142 (68.9)	149 (75.3)	0.361
	Yes	950 (19.5)	70 (29.2)	55 (30.7)		379 (27.6)	64 (31.1)	49 (24.7)	
Backache	No	4,296 (88.1)	185 (77.1)	142 (79.3)	< 0.001	1,101 (80.2)	155 (75.2)	160 (80.8)	0.415
	Yes	579 (11.9)	55 (22.9)	37 (20.7)		272 (19.8)	51 (24.8)	38 (19.2)	
Headache	No	4,429 (90.9)	196 (81.7)	150 (83.8)	< 0.001	1,210 (88.1)	175 (85.0)	175 (88.4)	0.415
	Yes	446 (9.1)	44 (18.3)	29 (16.2)		163 (11.9)	31 (15.0)	23 (11.6)	
Abdominal pain	No	4,617 (94.7)	209 (87.1)	151 (84.4)	< 0.001	1,280 (93.2)	191 (92.7)	183 (92.4)	0.896
	Yes	258 (5.3)	31 (12.9)	28 (15.6)		93 (6.8)	15 (7.3)	15 (7.6)	
Muscular pain	No	4,211 (86.4)	183 (76.3)	139 (77.7)	< 0.001	1,071 (78.0)	152 (73.8)	153 (77.3)	0.401
	Yes	664 (13.6)	57 (23.8)	40 (22.3)		302 (22.0)	54 (26.2)	45 (22.7)	
Fatigue	No	4,214 (86.4)	179 (74.6)	140 (78.2)	< 0.001	1,090 (79.4)	157 (76.2)	156 (78.8)	0.580
	Yes	661 (13.6)	61 (25.4)	39 (21.8)		283 (20.6)	49 (23.8)	42 (21.2)	
Psychological health problems	No	4,072 (83.5)	175 (72.9)	135 (75.4)	< 0.001	1,093 (79.6)	158 (76.7)	159 (80.3)	0.592
	Yes	803 (16.5)	65 (27.1)	44 (24.6)		280 (20.4)	48 (23.3)	39 (19.7)	
Stress	No	4,093 (84.0)	175 (72.9)	137 (76.5)	< 0.001	1,105 (80.5)	158 (76.7)	161 (81.3)	0.406
	Yes	782 (16.0)	65 (27.1)	42 (23.5)		268 (19.5)	48 (23.3)	37 (18.7)	
Sleeping problems	No	4,661 (95.6)	212 (88.3)	162 (90.5)	< 0.001	1,301 (94.8)	194 (94.2)	180 (90.9)	0.094
	Yes	214 (4.4)	28 (11.7)	17 (9.5)		72 (5.2)	12 (5.8)	18 (9.1)	
Anxiety	No	4,708 (96.6)	216 (90.0)	164 (91.6)	< 0.001	1,309 (95.3)	196 (95.1)	182 (91.9)	0.121
	Yes	167 (3.4)	24 (10.0)	15 (8.4)		64 (4.7)	10 (4.9)	16 (8.1)	
Depression	No	4,746 (97.4)	217 (90.4)	168 (93.9)	< 0.001	1,329 (96.8)	197 (95.6)	184 (92.9)	0.025
	Yes	129 (2.6)	23 (9.6)	11 (6.1)		44 (3.2)	9 (4.4)	14 (7.1)	

*Calculated by chi-squared test.

### Correlation of job insecurity of each contract type with work-related health problems

In order to examine the correlation between job insecurity and work-related health problems, multiple logistic regression was used according to contract type, adjusting for social demographic factors such as gender, age, education, income, occupation, smoking, and drinking, and work-related characteristics such as working hours, shift schedule, physical and chemical hazard exposure, and ergonomic risk factors. Analyzing only the temporary employees, those with higher job insecurity showed more correlation to having depression (OR 2.41; 95% CI = 1.24-4.68). Aside from that, there were no statistically significant associations between level of job insecurity and work-related health (Table 3). Unlike the temporary workers, however, the permanent employees with moderate job insecurity, compared to those with low, responded as having backache (OR 2.08; 95% CI = 1.49-2.90), headache (OR 2.19; 95% CI = 1.53-3.13), abdominal pain (OR 2.52; 95% CI = 1.66-3.81), muscular pain (OR 1.86; 95% CI = 1.35-2.58), fatigue (OR 2.12; 95% CI = 1.54-2.92), stress (OR 1.97; 95% CI = 1.44-2.68), sleeping problems (OR 3.04; 95% CI = 1.95-4.74), anxiety (OR 2.91; 95% CI = 1.82-4.65), and depression (OR 3.82; 95% CI = 2.34-6.23), all with statistical significance. The high job insecurity group also showed statistically significant results compared to those with low, reporting higher rates of backache (OR 1.78; 95% CI = 1.20-2.64), headache (OR 1.91; 95% CI = 1.25-2.93), abdominal pain (OR 3.27; 95% CI = 2.09-5.12), muscular pain (OR 1.68; 95% CI = 1.14-2.46), fatigue (OR 1.75; 95% CI = 1.19-2.57), stress (OR 1.65; 95% CI = 1.14-2.40), sleeping problems (OR 2.31; 95% CI = 1.33-4.02), anxiety (OR 2.33; 95% CI = 1.31-4.13), and depression (OR 2.26; 95% CI = 1.16-4.39). Additional analysis of the physical and/or psychological health problems and all types of health problems showed a higher odds ratio in the group with higher job insecurity than in those with low job insecurity (Table 4).

**Table 3:** Odds ratios (OR) and 95% confidence intervals for the association between job insecurity and work-related health problems in temporary workers

	**Crude OR**			Adjusted OR*		
	**Job insecurity**			**Job insecurity**		
	**Low**	**Moderate**	**High**	**Low**	**Moderate**	**High**
	**OR (95% CI)**	**OR (95% CI)**	**OR (95% CI)**	**OR (95% CI)**	**OR (95% CI)**	**OR (95% CI)**
All health problems	reference	1.18 (0.86-1.62)	0.83 (0.59-1.16)	reference	1.16 (0.83-1.61)	0.76 (0.53-1.09)
Physical health problems	reference	1.18 (0.86-1.62)	0.86 (0.61-1.22)	reference	1.14 (0.82-1.60)	0.78 (0.54-1.12)
Backache	reference	1.33 (0.95-1.88)	0.84 (0.66-1.40)	reference	1.23 (0.86-1.78)	0.85 (0.57-1.27)
Headache	reference	1.31 (0.86-1.99)	0.98 (0.61-1.55)	reference	1.22 (0.79-1.89)	0.83 (0.51-1.35)
Abdominal pain	reference	1.08 (0.61-1.90)	1.13 (0.64-1.99)	reference	0.97 (0.53-1.77)	0.96 (0.53-1.76)
Muscular pain	reference	1.26 (0.90-1.76)	1.04 (0.73-1.49)	reference	1.21 (0.85-1.73)	0.94 (0.64-1.36)
Fatigue	reference	1.20 (0.85-1.70)	1.04 (0.72-1.49)	reference	1.23 (0.86-1.78)	0.96 (0.66-1.42)
Psychological health problems	reference	1.19 (0.84-1.68)	0.96 (0.66-1.39)	reference	1.13 (0.78-1.63)	0.88 (0.59-1.30)
Stress	reference	1.25 (0.88-1.78)	0.95 (0.65-1.39)	reference	1.19 (0.83-1.73)	0.86 (0.58-2.10)
Sleeping problems	reference	1.12 (0.59-2.09)	1.81 (1.05-3.09)	reference	1.06 (0.56-2.04)	1.75 (0.99-3.10)
Anxiety	reference	1.04 (0.53-2.07)	1.80 (1.02-3.18)	reference	0.91 (0.45-1.84)	1.72 (0.94-3.14)
Depression	reference	1.38 (0.66-2.87)	2.30 (1.24-4.28)	reference	1.28 (0.59-2.78)	2.41 (1.24-4.68)

*Adjusted for gender, age, education, income, occupation, smoking, drinking, physical & chemical hazards, ergonomic risk factor, working hours, and work schedule.

**Table 4:** Odds ratios (OR) and 95% confidence intervals for the association between job insecurity and work-related health problems in permanent workers

	**Crude OR**			Adjusted OR*		
	**Job insecurity**			**Job insecurity**		
	**Low**	**Moderate**	**High**	**Low**	**Moderate**	**High**
	**OR (95% CI)**	**OR (95% CI)**	**OR (95% CI)**	**OR (95% CI)**	**OR (95% CI)**	**OR (95% CI)**
All health problems	reference	1.62 (1.21-2.15)	1.84 (1.34-2.54)	reference	1.57 (1.16-2.12)	1.81 (1.29-2.55)
Physical health problems	reference	1.70 (1.28-2.27)	1.83 (1.32-2.54)	reference	1.65 (1.22-2.23)	1.80 (1.28-2.55)
Backache	reference	2.21 (1.61-3.02)	1.93 (1.33-2.81)	reference	2.08 (1.49-2.90)	1.78 (1.20-2.64)
Headache	reference	2.23 (1.59-3.14)	1.92 (1.28-2.89)	reference	2.19 (1.53-3.13)	1.91 (1.25-2.93)
Abdominal pain	reference	2.65 (1.78-3.95)	3.32 (2.18-5.06)	reference	2.52 (1.66-3.81)	3.27 (2.09-5.12)
Muscular pain	reference	1.98 (1.45-2.69)	1.83 (1.27-2.62)	reference	1.86 (1.35-2.58)	1.68 (1.14-2.46)
Fatigue	reference	2.17 (1.61-2.94)	1.78 (1.23-2.56)	reference	2.12 (1.54-2.92)	1.75 (1.19-2.57)
Psychological health problems	reference	1.88 (1.40-2.23)	1.65 (1.17-2.34)	reference	1.90 (1.39-2.58)	1.69 (1.17-2.44)
Stress	reference	1.94 (1.45-2.61)	1.61 (1.13-2.29)	reference	1.97 (1.44-2.68)	1.65 (1.14-2.40)
Sleeping problems	reference	2.88 (1.89-4.37)	2.29 (1.36-3.84)	reference	3.04 (1.95-4.74)	2.31 (1.33-4.02)
Anxiety	reference	3.13 (1.99-4.91)	2.58 (1.49-4.47)	reference	2.91 (1.82-4.65)	2.33 (1.31-4.13)
Depression	reference	3.89 (2.45-6.20)	2.41 (1.28-4.54)	reference	3.82 (2.34-6.23)	2.26 (1.16-4.39)

*Adjusted for gender, age, education, income, occupation, smoking, drinking, physical & chemical hazards, ergonomic risk factor, working hours, and work schedule.

## Discussion

This study using the First Korean Working Conditions Survey data gathered in 2006, investigated the correlation between the level of job insecurity and work-related health problems among South Korean employees. In particular, stratifying according to contract type, analyses were performed to determined whether there were correlations between job insecurity and both the type of contract (temporary or permanent) and work-related health. On examination, a greater correlation with work-related health was found in the group with moderate or higher job insecurity in the permanent employees. The connection between health problems and job insecurity has been investigated in a variety of studies using either direct or indirect methods, and the majority of studies have reported that job insecurity has negative correlation to health [1,5,7-9,18,23-26]. In one study conducted with the employees of a major electronics company in South Korea, job insecurity was shown to affect psychological health in both male and female workers [12]. Another study on fire fighters and male employees of an automobile manufacturing corporation demonstrated that their depressive symptoms are linked to job insecurity [11]. Many other studies have reported similar results, such as a study on the relation between overweight and obesity, and job insecurity [8], and another on the association between job insecurity and subjective health perceptions and complaints of depressive symptoms. A study on insomnia incidence in relation to job insecurity also found similar results [9].

It is difficult to anticipate an appropriate action or provide timely social support for the phenomena that result from job insecurity, since it does not occur in an objective and distinctive manner such as actual unemployment or layoff [5]. In effect, in the case where job insecurity is experienced, it is often not easy for employees to find a way to work it out or find a solution, and in the end, the insecurity could materialize as chronic stress [3]. Our body, under a certain amount of stress, exudes hormones such as catecholamine, dopamine, serotonin, etc., following changes in the hypothalamus-pituitary-endocrine axis, limbic system, as well as autonomic nervous system-adrenal cortex [27]. These changes in hormones result in a variety of physical responses. For one, catecholamine can raise the blood pressure and heart rate, cause vasoconstriction of the vessels and hemodynamic changes, and activate platelet activities, to increase the risk of myocardial ischemia and thrombus formation. This is the reason exposure to stress could contribute to the onset or aggravation of cardiovascular diseases [28]. Sociopsychological factors linked with stress can also increase the degree of muscle tension and thus worsen the biomechanical tension in the musculoskeletal system causing an actual physical disability [29]. Stress also shows a connection to psychological well-being. It is thought to happen as a result of hormone changes such as dopamine and serotonin [30], or to those with a tendency to develop psychiatric diseases, who cannot overcome stress and succumb a full-blown malady [31].

Studies of job insecurity show a wide range of results for the incidence in employees according to the cohort setting (13.2%-51.2%) [5,32]. In our study, 11.6% of respondents at least moderate job insecurity, a result somewhat lower than in other reports. Such an outcome could be explained by the percentage of temporary workers included in a cohort [16,17], and we concluded that the direct comparison to the cohorts of other studies with different proportions of temporary workers would be impractical.

Our study results show that job insecurity is correlated to health in permanent employees, but not in temporary employees. This result is in accordance to many other reports already published that the job insecurity of permanent workers has a greater correlation to health than that of temporary ones [17]. One theory that explains the hypothesis that job insecurity has a greater impact on the health of permanent employees is the psychological contract theory. A psychological contract is a concept that with the expectations or sense of mutual obligation, one can form obligations to an organization one belongs to and fulfill it, to achieve the right to continue working for that particular organization [33]. In other words, the permanent worker forms a greater psychological contract than the temporary worker. In return for stability, they go as far as to tolerate expectations of the employer and obligations that follow, but as soon as they experience an incident that does not guarantee such stability within the employment arrangement, they tend to feel greater betrayal than the temporary employee, which then to becomes a more impactful stress factor in the form of job insecurity, to finally end in negative consequences to psychological and physical health and well-being [15]. Since the influence of job insecurity in accordance with the type of contract has not been clarified, however, and also whether the amount of work or nature of the work that is known to affect the health of the temporary workers has not been thoroughly analyzed, one cannot prematurely conclude that there is no connection between job insecurity and the work-related health of temporary employees per se.

In our research, job insecurity did not show a clear dose–response relationship in any of absence of related areas. We can assume from this result, that they do not feel either the presence or absence of security altogether but rather experience it on a continuum [3]. However, many previous studies have shown a dose–response curve [16,17], and this shows that there is a need for further studies in this area.

The strength of this study is having a large scale cohort population of 7,071, and that this is a sampling study that extends to the entire country, which can therefore be representative of the national population. In addition, when the survey was performed, well-trained surveyors were engaged, and systematic surveys were used so that random interpretation of the respondents could be prevented to minimize information bias. Also, another strength of this study was that, unlike other studies that focused on only a few symptoms or health problems, our study investigated a variety of work-related health problems.

Our research also has its limitations. Since our study is a cross-sectional study, proving a causal relationship between job insecurity and work-related health. In measuring job insecurity, only one clause was used, and not using the Occupational Stress Scale for Korean Employees [34] that is popularly used in the Korea or the scale of Ashford et al. that is used in many studies in other countries [35] can be considered as a drawback. There is not a generally accepted method of measuring such associations as yet [4], and different studies are using different methods of measurement [2]. The basic concept of such a variety of methods, however, has its cornerstone in understanding whether or not the current work position or employment status could be continued in the near future [4]. The clause in our study also has its foundation on such a concept, and the Fourth European Working Conditions Survey, which is the basis of the survey we have used in our study, has the same clause as a part of its investigation of job insecurity [36]. One meta-analysis also found that more than 30% of the studies it included used one clause for analysis [2], and our literature review also turned up many studies in which single-clause surveys were used. A question clause about work-related health problems also has its limitations in that as it is a self-reported questionnaire, there could be a disparity in the actual health of the workers from what they reported. Self-report questionnaires, however, are known to be a good predictive tool for the actual health factors, such as mortality [37]. There is also evidence that nationwide scale self-reported questionnaires in general show good reliability [38], and considering there are many studies that have used Working Conditions Survey data, which our study used as the health measurement method [9,39], using the survey in our study did not seem to be a problem. Future studies, however, measuring job insecurity and/or health problems, could use methods that have been proven valid and reliable to overcome the potential limitations of our study. Another limitation is that there is a gap between the known percentage of permanent and temporary workers and the percentage investigated in our study. In the same year that our study was carried out, Statistics Korea had actually determined that the percentage of permanent employees was about 64.5% through an economic activity survey [40], but the study we carried out found the percentage to be 74.9%, higher than that of Statistics Korea. It seems the difference seen here might be due to the different definitions of the contract type. Statistics Korea's economic activity population survey allows for overlap between types of temporary workers such as part-time work, employment by the hour, and irregular work, but in our study we have described temporary work in its exclusive form [22].

As it is expected that the number of employees experiencing job insecurity will increase consistently with ongoing changes in the labor market, there will be an increasing need for research on the health and working conditions in the future. Not only the use of surveys for subjective information, but objective information such as blood pressure measurement or taking blood samples for laboratory examinations is also needed, and research that aims to investigate methods to reduce job insecurity as well as the factors that cause it should be performed at the same time.

## Competing interests

The authors declare that they have no competing interests.

## Author’s contributions

WWL and JBP designed the research. MSK collected the data. WWL and KBM performed the statistical analysis. WWL, KJL, and JBP interpreted the data. WWL and KBM wrote the manuscript. All of the authors read and approved the final manuscript.
